# Recent Advances in Nanoparticle-Based Förster Resonance Energy Transfer for Biosensing, Molecular Imaging and Drug Release Profiling

**DOI:** 10.3390/ijms131216598

**Published:** 2012-12-05

**Authors:** Nai-Tzu Chen, Shih-Hsun Cheng, Ching-Ping Liu, Jeffrey S. Souris, Chen-Tu Chen, Chung-Yuan Mou, Leu-Wei Lo

**Affiliations:** 1 Division of Medical Engineering Research, National Health Research Institutes, Zhunan 35053, Miaoli County, Taiwan; E-Mails: ohnonancy@gmail.com (N.-T.C.); smallgi2002@gmail.com (S.-H.C.); cpliu2009@gmail.com (C.-P.L.); 2 Department of Chemistry, National Taiwan University, Taipei 10617, Taiwan; E-Mail: cymou@ntu.edu.tw; 3 Department of Radiology, The University of Chicago, Chicago, IL 60637, USA; E-Mails: sourisj@uchicago.edu (J.S.S.); c-chen@uchicago.edu (C.-T.C.)

**Keywords:** Förster resonance energy transfer, FRET, nanoparticle, biosensing, molecular imaging, drug controlled release, quantum dots, gold nanoparticles, mesoporous silica nanoparticles, upconversion nanoparticle

## Abstract

Förster resonance energy transfer (FRET) may be regarded as a “smart” technology in the design of fluorescence probes for biological sensing and imaging. Recently, a variety of nanoparticles that include quantum dots, gold nanoparticles, polymer, mesoporous silica nanoparticles and upconversion nanoparticles have been employed to modulate FRET. Researchers have developed a number of “visible” and “activatable” FRET probes sensitive to specific changes in the biological environment that are especially attractive from the biomedical point of view. This article reviews recent progress in bringing these nanoparticle-modulated energy transfer schemes to fruition for applications in biosensing, molecular imaging and drug delivery.

## 1. Introduction

Förster resonance energy transfer (FRET) is a process in which energy is transferred from an excited donor to an acceptor molecule, leading to a reduction in the donor’s fluorescence emission and an increase in the acceptor’s fluorescence emission intensities. Since the energy transfer efficiency is distance dependent, it can only occur over distances smaller than a critical radius, known as the Förster radius. FRET is therefore suitable for studying the distance between two molecules or two neighboring sites on a specific macromolecule, such as can be found during protein conformational change [1,2], protein interaction and enzyme activity [3–7].

Recently, a number of studies have incorporated nanoparticles into the design of the FRET system. Nanoparticles, such as quantum dots (QD), gold nanoparticles (AuNP) and upconversion nanoparticles (UCNP), possess unusual optical properties and can act either as a donor or a quencher, thereby enhancing FRET performance and providing flexibility in the selection of excitation wavelength. Some nanoparticles, like mesoporous silica nanoparticles (MSNs), have a different topological domain for separating donor and acceptor molecules, while others, like polymer-based nanoparticles, are able to self-degrade and thereby quench or de-quench the conveyed fluorophore. Such nanoplatforms can extend the utility and efficiency of FRET-based probes in exploring biological systems. In this review, we present the current status of nanoparticle-based energy transfer systems that use FRET for biosensing, molecular imaging and drug release profiling. More specifically, particle-dependent reviews on nanoparticle-mediated FRET can be found in recent literature [8–15].

## 2. Applications in Biosensing

FRET is recognized as a sensitive and reliable analytical technique, widely used in biological assays [16,17]. FRET is a more informative technique than simple fluorescence in such work, as it is very sensitive to nanoscale changes in distance between donor/acceptor moieties [18]. In FRET, the excitation energy of the donor’s electron is transferred to that of the acceptor’s via an induced-dipole movement interaction [19]. To improve FRET efficiency and analytical performance, considerable effort is expended upon the search for new energy donor/acceptor pairs. Traditional FRET organic dyes, as well as newer nanomaterials, like semiconductor-based QDs, rare-earth doped nanocrystals and AuNPs, can be used as FRET donors or acceptors, offering stronger FRET signals and more flexible sensing platforms in bioanalysis [20].

### 2.1. Quantum Dot-Based Biosensing

QDs serve as a great candidate for nanosensors in biological application, as they have many advantages over traditional/organic FRET pairs that include: high photostability, great emission intensity and significant resistance to photobleaching [21–24]. The large extinction coefficients and wide range of absorption wavelengths of QDs allow excitation of different emission wavelength QDs by a single excitation source, and QDs have precise, size-tunable emission wavelengths, determined by their quantum mechanical behaviors [23,24]. As a result of these characteristics, QDs are ideally suitable for long-term biosensors in tracking multiple bio-molecules. In addition to being directly used as fluorescent labels, QDs have been wildly exploited as efficient FRET donors for a wide variety of FRET acceptors. Their broad absorption and narrow emission spectra allow single-wavelength excitation of multiple donors and can avoid crosstalk with acceptor fluorophores. In addition, the spectral overlap between donors and acceptors can be adjusted by tuning the particle size during synthesis. QDs can also be coupled to multiple acceptor fluorophores for higher efficiency in energy transfer and can act as the support structure for biomolecules for imaging purposes or to simplify assay design.

**Figure 1:**
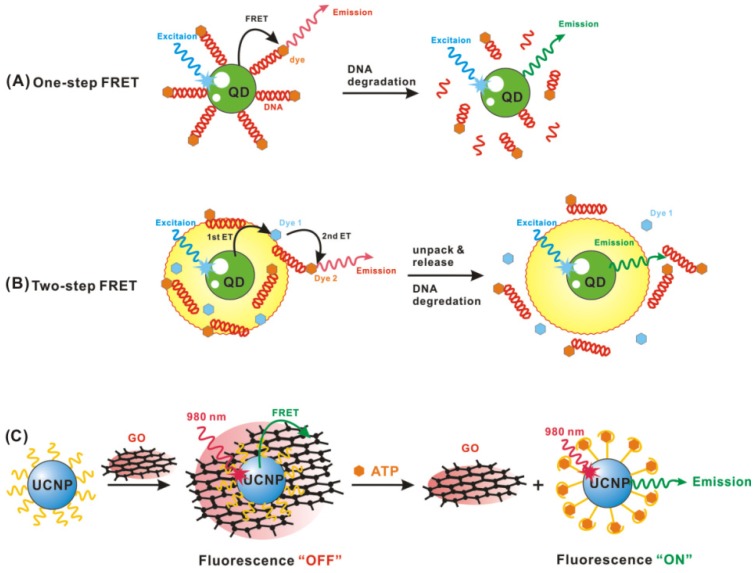

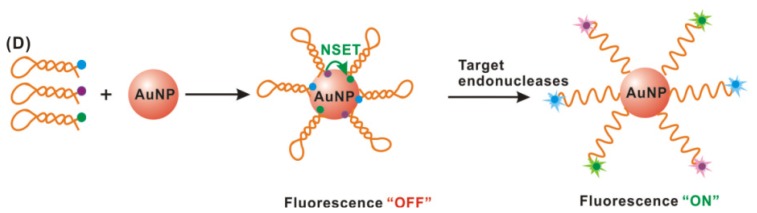
(**A**) Single-step FRET-based QD biosensors designed to probe DNA degradation. (**B**)The two-step QD-FRET system includes the first energy transfer from QD to Dye 1 in polymeric matrix and then the second energy transfer from Dye 1 to DNA-labeling Dye 2; (**C**) The FRET between ssDNA-UCNPs and GO for ATP sensing; (**D**) The enzyme-responsive multicolor gold nanobeacon for multiplex detection of endonuclease activity.

QDs have been used for biosensing applications employing a diverse array of energy transfer designs of single-step FRET systems (Figure 1A). For DNA detection, Leong’s group developed a single-step quantum dot-mediated FRET system to investigate the structural composition and dynamic behavior of plasmid DNA hybrid nanostructure *in vitro*[25]. Song *et al.* designed a positively-charged, compact QD–DNA complex for the detection of nucleic acids [26]. Such QD–DNA probes have the capability to detect the 200 nm H5N1 influenza virus oligonucleotide. Chen’s group developed a QD-aptamer (QD-apt) beacon that acts by folding-induced dissociation of a DNA intercalating dye, BOBO-3—demonstrated with label-free thrombin detection [27]. When mixed with a thrombin sample, the QD-apt beacon, BOBO-3 is competed away from the beacon, due to target-induced aptamer folding, leading to a decrease in QD FRET-mediated BOBO-3 emission—to quantify thrombin concentration.

Conventional assays for detection of endonuclease activity and inhibition, by gel electrophoresis and chromatography, are time-consuming, laborious, insensitive and costly. Recently, Huang *et al.* combined the high specificity of DNA cleavage reactions with the benefits of QDs (and ultrahigh quenching abilities of inter- and intra-molecular quenchers), to develop highly sensitive and specific nanoprobes for multiplexed detection of endonucleases [28]. Initially, the aminated QDs were conjugated with two sets of DNA substrates carrying quenchers, through direct assembly and DNA hybridization. When the nanoprobes were exposed to the targeted endonucleases, fluorescence was recovered via specific DNA cleavages, with the DNA fragments being released from the QD’s surface, along with the fluorescence quenchers. Thus, endonuclease activity was quantified simply by monitoring the change in the fluorescence intensity. Detection limits for *Eco*RI and *Bam*HI, using two different emission wavelength QDs, were at least 100-times more sensitive than traditional gel electrophoresis and chromatography approaches.

Beyond single-step donor-acceptor configurations, interest in incorporating QDs within multistep, biomolecularly-assembled FRET relays has grown [29–31]. Leong *et al.* demonstrated a two-step FRET system, which was constructed with a QD donor to the first acceptor of a nuclear dye (ND) (first energy transfer, E12) and the ND serving as a relay donor to the second acceptor Cy5 (second energy transfer, E23) [32,33] (see Figure 1A). When the nanocomplex begins to unpack and release intact pDNA, the E23 was off, thereby diminishing the emission of Cy5. With the intrinsic degradation of free pDNA, E12 ended. Thus, by monitoring the combinations of FRET-mediated emission from the ND and Cy5 with this two-step QD-FRET system, both polyplex dissociation and pDNA degradation within cells were sensed, simultaneously. For DNA hybridization detection, Rogach’s group has fabricated a hybrid nanostructure of CdTe conjugated polymers that exploits the broadband light harvesting and the FRET donor characteristics of QDs [34]. The conjugated polymer not only serves as a light harvesting antenna—to enhance the emission of QDs (the first-level FRET)—but also provides a positive-charged surface to enable negatively-charged dye-labeled DNA interaction This second-level FRET process, from QD to IRD700-labeled (an infrared fluorescence dye) DNA probe, provides a sensing platform to discriminate between complementary and non-complementary DNA. DNA hybridization was then quantified by the ratio of fluorescence intensity of IRD700 dye to that of QD.

Boeneman *et al.* have used QDs to function as potent initial FRET donors in a four-step FRET cascade along the length of DNA wires decorated with a series of fluorescent dye acceptors [35]. They conjugated multiple copies of DNA hybridized with four sequentially arranged acceptor dyes on the central QD, and demonstrated four consecutive energy transfers via both steady-state and time-resolved spectroscopic monitoring. However, achieving additional consecutive energy transfers has proven exceedingly difficult to obtain, even with the employment of QDs as optimal initial donors—generally due to the limited absorption capabilities of acceptor dyes. Given the advantages of using QDs as either an acceptor or a donor, it follows that QDs are best suited for use as intermediaries in FRET relay, where it can simultaneously function in both roles and enhance both energy transfer steps. However, the role of QDs as energy conveying intermediaries in FRET relays remains largely unexplored.

More recently, Algar *et al.* expanded the role QDs can play in FRET by demonstrating that QDs can function simultaneously as acceptors and donors within time-gated FRET relays [36]. Their bimolecular assemblies of Tb^3+^-complex-to-QD-to-Alexa Fluor 647 (A647) fluorescent dye provides a multistep FRET relay that includes the progressive time-gated Tb sensitization of the QDs via FRET step 1 (FRET1) and subsequent QD-to-A647 energy transfer via FRET step 2 (FRET2). Time-gating is essential to the observation of FRET1 and the subsequent energy relay via FRET2. Their time-gated photoluminescence (PL) lifetime measurements of both the Tb and its neighboring QD indicated that the Tb-to-QD FRET1 efficiency was *ca*. 94%–95%, equivalent to QD-to-A647 FRET2 efficiency. They demonstrated the utility of incorporating QDs into this type of time-gated energy transfer configuration in prototypical bioassays, for the monitoring of protease activity and nucleic acid hybridization. In this system, the Tb-to-QD FRET1 pathway sensitized time-gated PL and the QD-to-A647 FRET2 pathway provided a ratiometric, analytical signal, whose magnitude was proportional to the degree of biorecognition.

### 2.2. Rare-Earth-Doped Upconversion Nanoparticle-Based Biosensing

Organic dyes and QDs, however, do have some drawbacks in *in vitro* or *in vivo* FRET assays, due to the presence of strong autofluorescence signals that arise from cell and biomolecules at shorter wavelengths—a shortcoming less significant with UCNPs. UCNPs, display the unique property of converting low-energy NIR light to visible or hi gh-energy UV light. This process is based on sequential multiphoton absorption and energy transfer involving real, metastable, long-lived states [37]. The intra-particle energy transfer between two doping lanthanide ions, each in its excited state, leads to a shortening of wavelength emission. Depending on the particular lanthanide ion dopants employed, UCNPs typically function as energy donors and can provide a tunable emission that is compatible with a variety of acceptors that are commonly used in energy transfer nanocomposites for FRET-based biological/biomedical applications [38–43]. The use of UCNPs as donors can greatly improve the sensitivity and efficiency of FRET assays, since most biological materials do not absorb significant amounts of NIR light and the longer wavelength excitation circumvents the autofluorescence that arises at shorter wavelength excitation, the latter enhanced by the extremely narrow emission bands of lanthanide ions, spectrally separating biological background fluorescence from direct acceptor excitation.

Li’s group developed an upconversion biosensor based on the FRET between bioconjugated UCNPs and AuNPs; the first example of using the FRET techniques for a bioassay based on UCNPs [44]. In this work, they exploited the fact that the emission band of the biotinylated NaYF_4_:Yb, Er UCNPs overlaps well with the absorption band of the biotinylated AuNPs, thereby quenching fluorescence when both nanoparticles are in close proximity to one another. Consequently, they were able to conduct quantitative analysis of local avidin concentration, since the luminescence excited by NIR light was gradually quenched with increasing amounts of avidin. However, as Rantanen *et al.* have demonstrated [41], it is quite difficult to find an effective quencher to entirely quench the fluorescence of UCNPs, because only the emitters (rare earth ions) at or near the surface of UCNPs can be quenched. In previous studies, only AuNPs have been successfully employed as UCNP quenchers, with fluorescence quenching efficiencies of up to 80% [44,45].

Recently, graphite based structures with sp^2^ electronic hybrid orbitals and large conjugate planes have exhibited superquenching of fluorescence, and several FRET sensors have been developed employing graphene or graphene oxide as energy acceptors [46–52]. Liu *et al.* demonstrated, for the first time, that graphene oxide (GO) can act as an ultrahighly efficient quencher for ssDNA-functionalized UCNPs with which they constructed an extraordinarily sensitive and specific FRET-based biosensing platform successfully [53]. Since ssDNA can be strongly adsorbed onto the surface of GO via the strong π–π stacking effect between nucleobases of ssDNA and sp^2^ atoms of GO, the highly efficient fluorescence quenching of ssDNA-UCNPs can be considered as the direct consequence of efficient FRET between UCNPs and GO. Adenosine triphosphate (ATP) was selected as a proof-of-concept target to test the feasibility (see Figure 1B) and performance of a UCNPs-GO FRET pair-based biosensing platform. The observed sensitivity was more than two orders of magnitude higher than the detection limits of ATP in conventional assays (typically ~10 μm). Most recently, Wu *et al.* presented a new aptasensor for mycotoxins based on multiplexed FRET between multicolor BaYF_5_:Yb Er/Tm UCNPs as donors and GO as effective acceptors. Using this construct, they were able to detect two types of mycotoxins simultaneously and, in doing so, open up a new field of FRET system applications for a variety of targets [54]. Similar to the aforementioned UCNPs-GO FRET assay, Wang’s group developed the novel aptamer biosensor based on FRET using UCNPs and carbon nanoparticles (CNPs) as the energy donor-acceptor pairs [55]. They argued that the size of zero-dimensional CNPs is more comparable with biomolecules than two-dimensional graphene (or graphene oxide)—with a relatively large plane and fluorescence quenching ability to be expected, due to the sp^2^ electronic structure’s similarity with that of graphene.

### 2.3. Gold Nanoparticle-Based Biosensing

AuNPs are excellent FRET-based quenchers because of their large extinction coefficients and broad energy absorption bandwidth in the visible range. Fluorescence quenching-based turn-on assay is one of the most important applications of FRET-based techniques. In this system, the fluorescence of the donor can be effectively quenched by the acceptor in the absence of the assay target. The quenched fluorescence is “turned-on” upon the addition of targets, with the restored fluorescence intensity being proportional to the concentration of targets. The most typical application of AuNPs in FRET-based assays is the labeling of molecular beacons [18]. Molecular beacons are hairpin shaped molecules bearing an internally quenched fluorophore, whose fluorescence is restored when they bind to a target nucleic acid—the action of which separates the fluorophore from the quencher. For example, a novel molecular beacon, developed by Xu and Hepel, based its detection of glutathione (GSH) and cysteine (Cys) upon Hg^2+^-induced self-hybridization of the beacon strand [56]. In their system, they situated a fluorophore and a quencher at the ends of the stem that contained a T-T mismatch for complexation of a Hg^2+^ cation. In a very selective coordination of Hg^2+^ to GSH/Cys, the fluorophore/quencher distance increases concomitantly with the dehybridization and dissociation of the beacon stem T-Hg^2+^-T, due to extraction of Hg^2+^ ions, thereby switching the molecular beacon on. This approach enables a wide concentration range of functionality (4 to 200 nm), with detection limits of 4.1 nm for GSH and 4.2 nm for Cys.

Huang’s group reported a highly sensitive and selective assay based on an enzyme-responsive multicolor gold nanobeacon for the multiplex detection of endonucleases [57] (see Figure 1C). Initially, each of three hairpin DNA reporters was labeled with a fluorescent dye that responds to one of three different endonucleases, co-assembled at the surface of AuNPs (15 nm). Fluorescence quenching occurred due to the nanosurface energy-transfer (NSET) effect, as this assembly brings the dyes into very close proximity with AuNPs. Upon binding with the target endonucleases, specific DNA cleavages occur in the nanobeacon, following with DNA fragments being released from the surface of AuNPs along with the fluorescence dye. Thus, measurement of fluorescence recovery provided the basis for a quantitative measurement of endonuclease activity.

Other groups have employed AuNP-based FRET monitors for the detection of DNA hybridization and protein with varying of the DNA length [58–61]. Cheng *et al.* investigated the size and distance dependence of fluorescence near gold nanoparticles in a homogeneous DNA assay, leading to their proposal of a sensitive DNA probe [60]. In this system, a closed hairpin resulted in the contact of fluorophores and AuNPs, leading to an efficient energy transfer (*i.e.*, fluorescence quenching). Upon DNA hybridization, the DNA stretches, thereby restoring fluorescence. The size and distance dependences were not empirically studied and, thus, inferred via finite difference time domain (FDTD) simulations and the Gersten-Nitzan model. Based upon these experimental and numerical studies, Ceng *et al.* reported over 96.8% quenching efficiency for all particle sizes and optimal fluorescence using 100 nm AuNPs with 35 bp DNA strands. This is the first study of the quantum efficiency and enhancement factor in the GNP-DNA-dye homogeneous system with AuNPs larger than 20 nm in diameter. The thorough investigations and methodology developed by them has helped establish a database for homogeneous DNA sensing.

Bovine serum albumin (BSA) bioconjugate nanoparticles are of great importance because of their potential applications in biomedicine and, more importantly, as biosensors. Bioconjugate nanoparticles have been used as an efficacious approach for increasing colloidal stability [61,62] and enhancing biocompatibility in a variety of nanoparticle systems [63–65]. Recently, the interactions of AuNPs and bovine serum albumin-gold nanoconjugates (BSA-AuNPs) with CdSe QDs were investigated by Mandal’s group [66]. They employed steady-state and time-resolved fluorescence spectroscopic techniques to analyze fluorescence quenching of QDs based on FRET in the presence of BSA-AuNPs. However, they observed that in the presence of only AuNPs, the fluorescence quenching of QDs is primarily static in nature. They postulated that QDs and AuNPs, being separated by short distances in the bionanoconjugate system, facilitated energy transfer, rather than the formation of ground-state complexes in which GNPs directly contact QDs. The effect of QDs on the conformation of BSA-GNPs was also examined and analyzed (by circular dichroism spectra), indicating that the secondary structure of the bionanoconjugate undergoes marginal changes. This type of interaction between QDs and GNPs in a protein-conjugate provided a new insight for design and the development of FRET-based bionanosensors.

## 3. Application of Molecular Imaging

When confronted with injury, disease or even therapeutic intervention, dramatic local and systemic changes (e.g., changes in pH, oxygenation, enzyme activity, *etc.*) in the human body’s microenvironments occur. Knowledge of where and to what extent the microenvironment has deviated from its norm can be of significant clinical relevance. To detect and image these changes *in vivo*, a number of “smart” nanoprobes have recently been developed [67,68]. Nanoparticle-based fluorescence probes have been widely used as optical contrast agents for a variety of biological applications that include bioimaging and diagnostics. Nanomaterials, like QDs, can have spectacular optical properties, such as high quantum yield and a tunable narrow emission band, that suggest their being excellent energy donors in FRET-based imaging probes. Polymer-based nanomaterials, by comparison, have relatively larger loading capacities and the ability to self-degrade, whereby they can quench/de-quench contained fluorophores. And as noted earlier, UCNPs, unique to converting longer wavelength NIR light to shorter wavelength UV or visible light, circumvent one of the primary impediments encountered with biomaterials: their strong autofluorescence for wavelengths less than 600 nm. In the following paragraphs, we will focus on progress made in the developments of subsets ofnano-based FRET probes known as “activatable” or “smart”; in contrast to agents that achieve very high target-to-background signal ratios compared to conventional “always on” imaging agents and enable imaging intracellular targets.

### 3.1. Quantum Dot-Based Molecular Imaging

QDs have high quantum yields and tunable narrow emission bands of QD, making them especially attractive as efficient energy donors in FRET-based probes. In recent reports, FRET between quantum dots and organic fluorophores has been exploited to yield constructs suited to imaging of a wide array of fundamental biological processes, as well as to create biosensors [68–70]. In the majority of these studies, the organic fluorophores were adsorbed onto the surface of QDs via proteins, peptides or polymers. Numerous investigators have also incorporated QD-based FRET technology in their attempt to track and quantify enzyme activity [3–7], protein conformation change [1,2] and DNA condensation/decondensation status [71]. In the following, we summarize QD-based molecular imaging probe developments that utilize FRET to sense *in vitro/in vivo* biological mechanisms.

Enzymes play key roles in physiology and pathology. The detection and quantitative measurement of enzyme/substrate interactions within living cells are of critical importance to our understanding of cell physiology and pathophysiology. Among the plethora of enzyme types, a number of them are proteolytic enzymes that degrade specific peptide sequences located on/within proteins. This sequence-specific recognition and cleavage has been successfully applied to design the bimodal “on” and “off” QD-based FRET beacon. Kim *et al.* developed a proteolytic-activated FRET pair for sensing the activity of MMP-7, a protease involved in the degradation of extracellular matrix during cancer cell metastasis. In their molecular beacon, The MMP-7 cleavable peptide sequence RPLALWRSK was incorporated between QDs and the fluorescence molecular dye TAMRA. While the PL intensity of the donor QD is quenched by TAMRA dyes, the addition of MMP-7 causes cleavage of the peptide substrate, which results in recovery of the PL of the QDs and a simultaneous decrease in TAMRA light emission [7]. Kim *et al.* employed an AuNP as a quencher for this FRET probe in a multiplexed assay system of MMPs and their inhibitors, measuring the energy transfer between QDs and AuNP. In this system, while the PL of donor QDs that had been immobilized on a surface was quenched via the presence of proximal AuNPs (energy acceptor), the protease activity caused modulation in the efficiency of the energy transfer between the acceptor and donor, thus enabling protease assay. In comparison to the QD-dye system, the conjugate of the QD-AuNP not only gave rise to the higher energy transfer efficiency, but also permitted it, with the use of different sizes (thus, a different wavelength emission) of QDs, multiplexed assay with MMP, caspase and thrombin. Such multiplexing arises from the broad absorption spectra of AuNPs and the narrow emission spectra of QDs: a single AuNP could be used as a common energy acceptor from several different size/wavelength QDs [5]. QD-based FRET probes have also been successfully employed in cell-based visual assays for protease-targeted, anti-retroviral drug discovery. Song *et al.* demonstrated a QD-peptide probe that was prepared via simple one-step electrostatic conjugation for detecting HIV-1 protease (HIV-1 PR) activity in living cells *in vitro* with picomole sensitivity [4]. Like protease activity, kinase activity has also been studied using QD-FRET probes. Kinases are of critical importance in cell signaling and cancer biology for the recognition and phosphorylation of specific proteins. Ghadiali and his co-workers demonstrated that self-assembled peptide-quantum dot conjugates can serve as surrogate substrates in a simple homogeneous assay for protein kinase activity. In their work, fluorophore-labeled antibodies were able to bind to the QD-peptide only if the kinase phosphorylated on the peptide [72].

QD-based FRET probes can also be used for real-time biodynamics imaging. Imaging of the change in pH within live cells is crucial to our understanding and quantification of cellular metabolism, disease and response to therapy. FRET pairs comprised of a QD donor and pH-sensitive fluorescence protein (FP) acceptor have been developed, wherein the environmental sensitivity of the acceptor fluorophore modulates the emission intensity of the donor. Dennis *et al.* proved that the QD-based ratiometric pH sensor yields dramatic improvements in imaging sensitivity and photostability compared to BCECF, the most widely used fluorescent dye for pH imaging. They also found that FRET between the QD and FP modulates the FP/QD emission ratio, exhibiting a 12-fold change between pH 6 and 8—well suited for visualization of the acidification of endosomes in living cells [73]. Cytotoxic entities, such as fluoride ions (F^−^), can be detected within living cells by the QD-based FRET systems. A high sensitivity and selectivity fluoride ion (F^−^) nanoprobe has been developed based upon the FRET between QDs and AuNPs through the formation of cyclic esters between phenyl boronic acid and the diol. In the presence of F^−^, the boronate ester (a “hard acid”) strongly reacts with F^−^ (a “hard base”), converting the boronate ester into trifluoro borate. This conversion, which causes results in the cleavage of the QD-AuNP linker, results in the fluorescence recovery of the quenched QDs. With its low 50 nm detection limit of the F^−^ and low cytotoxicity, this nanoprobe provides a viable alternative to current practices for the detection of F^−^ in biological and environmental samples [74,75].

**Figure 2:**
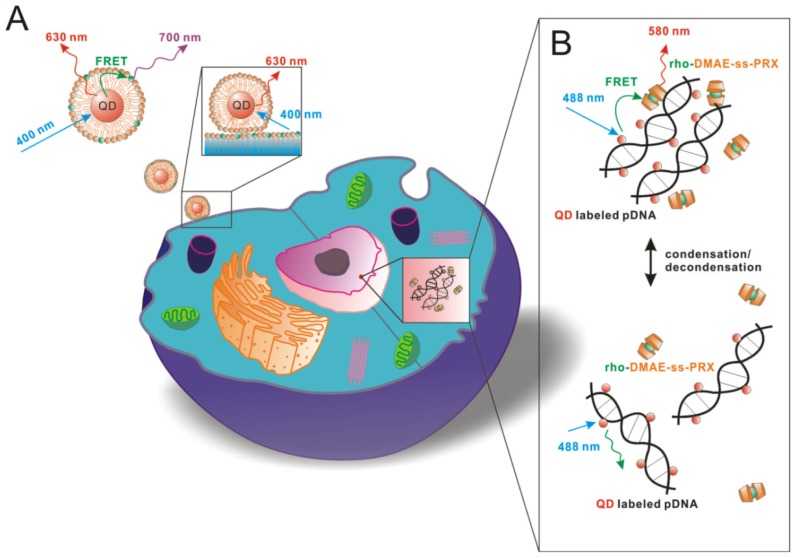
(**A**) Schematic representation of the lipid-exchange between Cy5.5-labeled lipids of QD-HDL-Cy5.5 and lipids of the cell membrane, as well as its effect on FRET; (**B**) Quantitative analysis of the condensation/decondensation status of pDNA by FRET between QD-labeled pDNA and rhodamine-labeled DMAE-ss-PRX.

The dynamic, intracellular status of living cells, such as lipid exchange or DNA condensation, can also be monitored by the QD-based FRET [71,76] (Figure 2). Skajaa’s group developed a QD-HDL FRET probe labeled with the near-infrared dye Cy5.5. They used this construct to investigate lipid-exchange dynamics, cellular interactions and nanoparticle disassembly (Figure 2A), demonstrating a methodology to follow lipid exchange between HDL and other lipidic nanoparticles, and confirming the stabilizing features of the apoA-I [76]. Shaheen *et al.* established a technique for imaging the nuclear condensation/decondensation status of pDNA in nuclear subdomains using FRET between QD-labeled pDNA (as donor) and rhodamine-labeled polycations (as acceptor) (Figure 2B). FRET strengthened when pDNA and polycation molecules were tightly bound and decreased as donor/acceptor separation grew. pDNA in the condensation/decondensation status in heterochromatin or euchromatin were quantified based on the pixel area of the signals derived from the QD and rhodamine [71].

Distance and orientation between FRET pairs significantly affect the efficiency of energy transfer. Boeneman’s group designed a QD-based FRET probe to specifically examine the factors that influence FRET energy transfer efficiency. They compared the architecture that results from using two common self-assembly chemistries for the attachment of DNA to QDs: (1) polyhistidine peptide-DNA assembly onto QD surfaces and (2) biotinylated DNA attachment to streptavidin-coated QDs. Complementary dye-labeled DNAs were hybridized to different positions on the DNA in each QD configuration, and QD/dye (donor/acceptor) separation distances were probed with FRET. The DNA modified with the polyhistidine self-assembles onto QDs, with its structure extended out from the QD-PEG surface, as predicted in numerical studies. By contrast, the random orientation of streptavidin on the surfaces of QDs resulted in DNA with a wide variety of possible orientations relative to the QD that cannot be controlled during self-assembly. These findings suggest that selection of attachment chemistry between FRET pairs strongly influences the resulting composite architecture [77].

### 3.2. Polymer-Based Molecular Imaging

**Figure 3:**
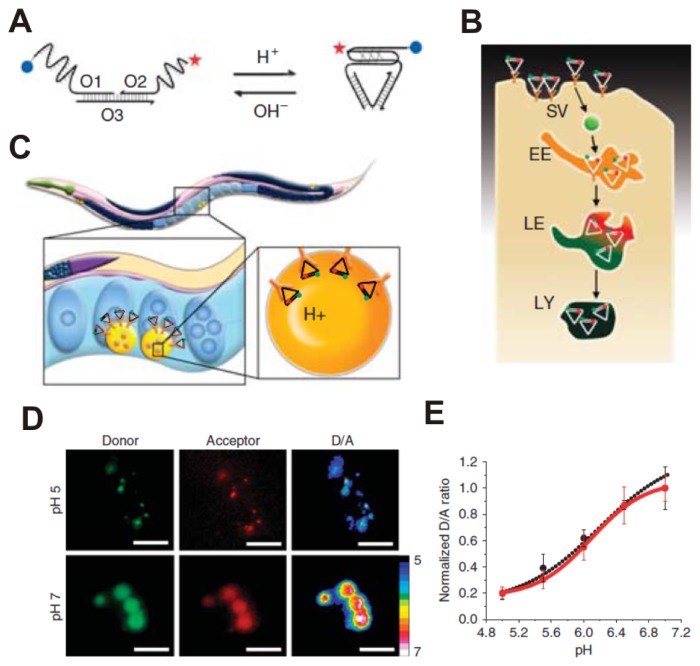
The “I-switch” DNA nanomachine recapitulates its pH-sensing properties *in vivo.* (**A**) the structure and working of an I-switch; (**B**) I-switch uptake in coelomocytes postinjection in *C. elegans*; (**C**) Stages in endosomal maturation of receptor-mediated endocytosis of a ligand; (**D**) donor channel, acceptor channel and respective pseudocolor D/A images of wild-type coelomocytes labeled with I-switch and clamped at pH 5 and 7. Scale bar, 10 ìm; (**E**) pH calibration curve of I-switch *in vivo* (red trace) and *in vitro* (black trace) showing normalized D/A ratios *versus* pH. Error bars indicate s.e.m. (*n* = 25 cells, ≥50 endosomes). Figures reproduced with permission from Macmillan Publishers [79].

Spatial and/or temporal information about the endocytosis status is of tremendous utility in the development of drugs. Toward this end, Krishnan’s group recently developed an intracellular pH imaging technique that employs DNA nanomachines, termed “I-switches” (Figure 3). I-switches are nucleotide assemblies that switch between rational molecular conformations in response to external stimuli. The combination of pH-responsive nucleotide assemblies and multiple color fluorophores enables pH-triggered FRET within living cells. With I-switches, spatial and temporal pH changes associated with endosome maturation can be detected and imaged. The fine performance of I-switches inside live cells holds great potential for them in diagnostics and targeted therapies [78]. In these studies, I-switches have also been used to monitor *in vivo* endosomal maturation in receptor-mediated endocytosis (Figure 3A–C). Krishnan’s group employed the transparent nematode *Caenorhabditis elegans* to map spatiotemporal pH changes associated with receptor-mediated endocytosis in wild-type, as well as mutant, worms. Figure 3D shows the wild-type *Caenorhabditis elegans* labeled withI-switches compared with the *in vitro* (black) and *in vivo* (Red) pH calibration curves (Figure 3E). The I-switch demonstrated autonomous function within the organismal milieu in a variety of genetic backgrounds [79].

More recently, designers of polymer-based FRET nanoprobes for molecular imaging have shifted their operations towards NIR wavelengths, largely for *in vivo* applications [80]. Wagh *et al.* report the rational design of FRET-based polymeric nanoparticles for NIR and FRET imaging. The particles were assembled from diblock copolymers, which were also encapsulated with both the donor, 1,1-dioctadecyl-3,3,3,3-tetramethylindodicarbocyanine (DiD), and the acceptor, 1,1-dioctadecyl-3,3,3,3-tetramethylindotricarbocyanine (DiR), fluorophores. This FRET system generates a large Stokes shift (>100 nm) and, thus, can reduce background autofluorescence in live animals.

### 3.3. Rare-Earth-Doped Upconversion Nanoparticle-Based Molecular Imaging

Upconversion nanoparticles (UCNP) have become the prominent FRET materials for biological imaging, especially for the *in vivo* imaging system [81–88]. Owing to the intense visible emission from these nanoparticles under near-infrared excitation, the latter of which is less harmful to biological samples and has greater sample penetration depths than conventional ultraviolet excitation, enhances their prospects as luminescent stains in bioimaging. In addition to their small physical dimensions and biocompatibility, UCNPs can be easily coupled to proteins or other biological macromolecules and used to sense/target specific molecules in small animal models of human disease. Xiong’s group developed cRGD-UCNP constructs and injected them into athymic nude mice bearing subcutaneous U87MG tumors overexpressing ávâ3 integrin. The progress of cRGD–UCNP tumor targeting was monitored by *in vivo* imaging, with the majority of nanoplatforms reaching their goal by 4 h post-injection. Of particular note is that *in vivo* imaging showed great contrast (signal-to-noise ratio ~24) between the tumor and background [86].

**Figure 4:**
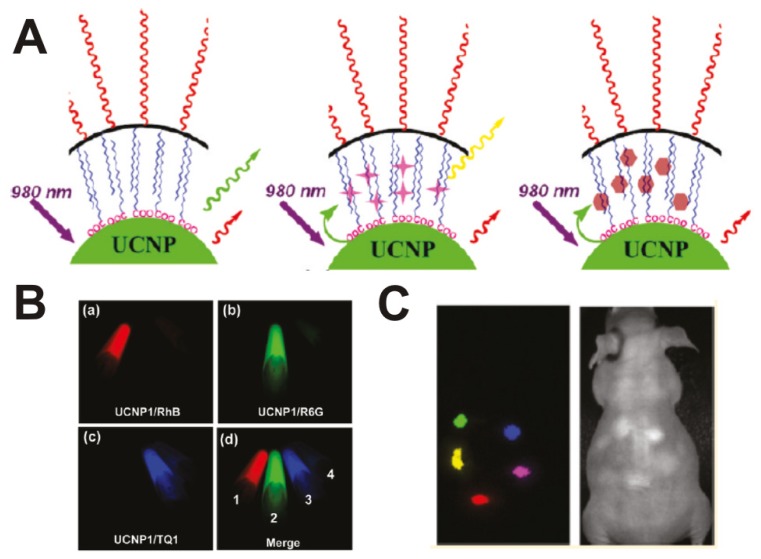
UCNP-based LRET system. (**A**) A scheme of the UCNP-LRET system; the hydrophobic layer between the inorganic nanoparticle surface and the external PEG coating allows adsorption of organic dye molecules on UCNPs; (**B**) Multicolor UCL images of three UCNP-dye complexes and a mixture of the three. The images were obtained using a Maestro *in vivo* imaging system after spectral unmixing; (**C**) *In vivo* multicolor UCL images of a nude mouse subcutaneously injected with five colors of UCNPs solutions. Merge-UCL (left) and white light (right). Figures reproduced with permission from The American Chemical Society [88].

A chromophoric ruthenium complex-assembled nanophosphor (N719-UCNPs) has been developed as a highly selective water-soluble probe for upconversion luminescence sensing and bioimaging of intracellular mercury ions by Li and his co-works [87]. The design strategy of Hg^2+^-selective chromophoric upconversion nanosystem N719-UCNPs is based on the Hg^2+^ modulating energy transfer degree from upconversion luminescence (UCL) emission of the nanophosphor to the absorbance of the complex N719. Using the ratiometric upconversion luminescence as the detection signal, the detection limit of Hg^2+^ for this nanoprobe in water was only 1.95 ppb, lower than the maximum level (2 ppb) of Hg^2+^ in drinking water set by the United States EPA. Importantly, N719-UCNPs have also proven capable of monitoring changes in the distribution of Hg^2+^ in living cells by UCL bioimaging. More recently, Cheng *et al.* developed multicolor *in vivo* imaging of UCNPs with emissions tuned by luminescence resonance energy transfer (LRET) [88] (Figure 4A). In this platform, several types of organic fluorescent dye and quencher molecules are physically attached to the surface of PEGylated UCNPs via hydrophobic interactions. The formed supramolecular UCNP dye complexes show tuned visible emission spectra owing to the LRET from nanoparticles to the organic dyes under near-infrared (NIR) excitation and can be well separated in multicolor imaging after spectral decovolution/unmixing (Figure 4B). *In vivo* five-color upconversion luminescence imaging using UCNPs and dye-loaded UCNPs have been successfully used in proof-of-concept animal experiments via spectral deconvolution (Figure 4C)—providing a facile and flexible method to modulate the upconversion luminescence (UCL) spectra of UCNPs for *in vivo* multicolor UCL imaging.

## 4. Application in Dynamic Monitoring Drug Releasing

Nanoparticle-formed drug delivery systems (DDSs) have been of considerable interest in the recent years because of their great potential to improve the efficacy of the delivery of traditional small molecular drugs [89]. Consequently, a wide variety of DDSs have been developed in a number of different formulations that include liposomes, micelles, gelatin and inorganic nanoparticles [90–95]. Polymeric nanoparticles can encapsulate drugs and release them in a regulated fashion through surface erosion of the nanoparticles, diffusion of the drug through the polymer matrix or swelling followed by diffusion [96–98]. The rigid matrix of inorganic nanoparticles, however, does not generally allow for encapsulation and subsequent release of active molecules for conventional drug delivery. Instead, drug molecules can be linked to, or combined with, nanoparticles for targeted delivery [99–103]. For “smart” stimulation-responsive release of drugs, scientists design a variety of “switch” linkages that can be activated by external stimuli, such as light (trans-cis molecules) [104], pH value (hydrazone) [105], redox (disulfide bonding) [106] or enzyme response [107]. To evaluate the therapeutic benefit of these various DDSs, one needs to monitor drug release from the delivery system *in vivo*, or, at minimal, in living cells. Especially interesting and informative is the potential of obtaining an *in vivo* drug release status in real-time from nano DDS via FRET mechanisms, owing to the high spatial content of such signals. Our review the recent advances in the dynamic monitoring of drug release of nano DDSs will thus focus on their use of FRET techniques.

In recent years, a number of “theranostic” nanoparticles comprised of targeted dual diagnostic and therapeutic moieties [108–115]. Contrast enhancement arises from the inclusion of optical or radioactive species that either emit signals spontaneously or upon excitation by an external source. To monitor the dynamics of therapeutic drug release, environmentally-induced changes in the physical or chemical interaction between signal source and therapeutic payload could be used. In the case of using FRET techniques to monitor drug release, several conditions must be satisfied in the preparation of nano DDS: (i) Two different but spectrally compatible fluorophores are incorporated into the core of nano DDSs to serve the energy donor and acceptor. Because FRET interactions are very sensitive to donor-acceptor separation distance, FRET will occur only in DDSs in which carriers are in intimate contact with donor/acceptor pairs; (ii) One of FRET-pair fluorophores must be discharged from the carrier with the drug and diffuse outwards, while the nanoparticle carriers break down upon external stimuli. Such a scenario increases the average distance between the donors and acceptors and, thus, decreases the FRET signal. In the next few paragraphs, we discuss recent developments in the application of FRET to monitor drug release from organic and inorganic nano DDS.

### 4.1. Quantum Dot-Based Drug Delivery System

**Figure 5:**
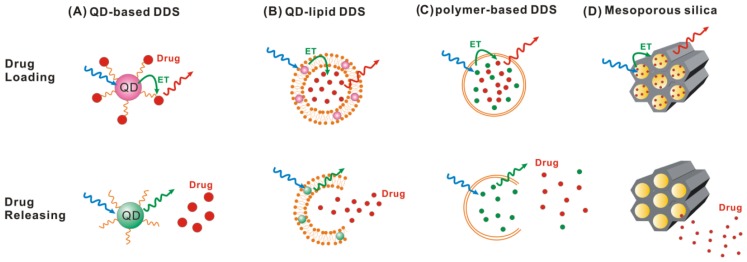
FRET monitoring of drug release from a variety of DDS, including (**A**) quantum dot-, (**B**) quantum dot-lipid, (**C**) polymer-, and (**D**) mesoporous silica-based delivery systems.

QD-based drug delivery systems have a few general attributes in common: (i) the nanoparticle surface is functionalized with targeting ligands for specific delivery; (ii) the drug molecules are released at tumor cells after being triggered externally or by local environmental factors; and (iii) the surface of QDs are covered with a long-lasting biocompatible polymer to prevent immune attack, premature excretion and to prolong circulation. Owing to these characteristics, QDs provide a versatile platform for engineering traceable drug delivery systems with the potential for improving pharmacological treatment of cancers. QDs also have a great photostability and resistance of photobleaching, as compared to conventional organic fluorophores. Therefore, numerous investigators have sought to use QD as the drug carrier and to monitor the dynamics of drug release from QDs using FRET (Figure 5A) [116,117]. Indeed, QDs have been wildly popular as efficient FRET donors because of their photoluminescence characterized with broad absorption and narrow emission (minimal spectral cross-talk) that makes the selection of the acceptor easy.

Bagalkot’s group has developed QD-based theranostics for simultaneous imaging and therapeutic application [118]. Multifunctional QDs were conjugated with targeting RNA aptamers that recognize the extracellular domain of the prostate-specific membrane antigen, enabling preferential targeting and imaging of prostate cancer cells (LNCaP). Instead of just targeting, the double-stranded RNA aptamer was additionally used to intercalate anticancer drugs doxorubicin (Dox) into the cancer cell’s DNA for therapeutic activity. In this bi-FRET (dual donor/quencher) system, the QD fluorescence was quenched by Dox and the fluorescence of Dox was quenched by the RNA aptamers. While the Dox was loading, both QD and Dox fluorescence were turned “OFF” through bi-FRET. When the QD-Apt complex was treated with prostate cancer cells and Dox was gradually released, both QD and Dox fluorescence switched to “on”. This multifunctional QD-Apt-Dox delivered Dox to LNCaP cells much more efficiently than non-targeted PC3 prostate cancer cells, with the efficacy of Dox delivery monitored by FRET simultaneously.

QDs have also been employed with more complex bio-molecules, such as short interfering RNA (siRNA) [119–122]. QDs, appropriately surface-functionalized with cationic moieties, make ideal siRNA carriers, as they not only engender these gene therapy drugs with physiological stability and target specificity, but also with the ability to be optically tracked. Lee and his co-workers reported a siRNA/QD-PEI complex for the analysis of intracellular siRNA uptake, as well as the quantitative evaluation of siRNA unpacking from the cationic QD carriers by FRET analysis [123]. The authors used flow cytometry to study the FRET interactions between cyanine dye labeled siRNA (Cy5-siRNA) and cationic QD carriers. QD-PEI was further modified with a protein transduction domain (PTD) from protein transduction domain peptide (Hph-1). The siRNA/QD-PEI complexes with and without Hph-1 exhibit different unpacking kinetics of Cy5-siRNA and intracellular uptake—demonstrating that siRNA/QD-PEI complexes can be utilized for intracellular trafficking of siRNA release to maximize the silencing of targets.

Polymers, such as liposomes, or polysaccharides have been widely used to construct hybrid vesicles in which QDs are typically incorporated into the lipid/carbohydrate to enable fluorescence tracking. QD-liposomes (QD-L) have been developed as a theranostic DDS for the encapsulation of Dox or other water-soluble drug molecules (Figure 5B) [124–126]. Weng *et al.* incorporated covalently-conjugated hydrophilic, polymer-functionalized QDs into the outer layer of liposomes with the polyethylene glycol chains coating and then loaded Dox into the core of the vesicles [127]. Tian *et al.* designed drug-loaded QD-L-Dox hybrid vesicles as theranostic platforms [128]. A hydrophilic fluorescence dye (carboxyfluorescein) was encapsulated within the internal aqueous phase of the QD-L hybrids. This QD-L-Dox hybrid was used to measure the difference in kinetics of drug release in the buffer and serum, a difference that depended on the type of lipid used in hybrid synthesis. DSPC-type QD-L hybrids have shown the highest stability of all formulations studied, with minimal carboxyfluorescein leakage and no change in size over a period of three weeks. Noteworthy is the fact that Dox was loaded into QD-L hybrids using simple osmotic gradient techniques, with at least 97% loading efficiency. Changes in the fluorescence (emission) spectrum of Dox were simultaneously observed as Dox was released from the QD-L-Dox hybrid vesicles. Overall, the drug-loaded QD-L-Dox hybrid vesicles provide a promising multifunctional delivery vector capable of transporting combinations of therapeutic and diagnostic moieties. Gopalakrishnan *et al.* also developed a hybrid QD-L system and used the fluorescence correlation spectroscopy (FCS) to monitor the diffusion of QDs as their conveying liposome fused with the targeted cell’s plasma membrane [124]. The diffusion of QDs in the plasma membrane and in the lipid bilayer was nearly identical, but that of QDs in small unilamellar vesicle solution showed much slower diffusion.

Wu *et al.* reported polysaccharide-based hybrid nanogels that can form the functional building blocks of a single nanoparticle system capable of optical pH-sensing, cancer cell imaging and controlled drug release [129]. These hybrid nanogels were developed by *in situ* immobilization of CdSe QDs within the interior of dual responsive (pH and temperature) hydroxypropylcellulose-poly (acrylic acid) (HPC-PAA) semi-interpenetrating polymer networks. The HPC-PAA-CdSe hybrid nanogels combine to form a strong trap emission at 741 nm—to sense the physicochemical environment in a pH-dependent manner. On the other hand, a visible excitonic emission at 592 nm can be used for mouse melanoma B16F10 cell imaging. The hybrid nanogels also provide excellent stability as a drug carrier for a model anticancer drug, temozolomide. They offered a pH-triggered sustained-release of the drug molecules in the gel network.

### 4.2. Polymer-Based Drug Delivery System

Chen *et al.* developed a polymer-based micelle for drug delivery that revealed the release of hydrophobic molecules by FRET [130]. The dual-labeled polymeric micelles were prepared using fluorescently-labeled copolymers that assembled in solution around hydrophobic fluorescent probes that consequently comprised the micelle’s core—under the assumption that the cellular uptake of core hydrophobic probes would be much faster than that of labeled micelle copolymers. Both lipophilic tracers of DiI and DiO dyes were loaded into monomethoxy poly(ethylene glycol)-block-poly(d, l-lactic acid) micelles to form FRET pairs (Figure 5C). Real-time tracking of micellar release of core hydrophobic probes into the extracellular space was accomplished by FRET imaging and spectroscopy. FRET efficiency calculations revealed that polymeric micelle release of hydrophobic molecules took place via a membrane-mediated transport mechanism. Chen *et al.* also observed a decrease in the FRET signal of plasma membranes when studying the latter’s interactions with paclitaxel-loaded polymeric micelles. From these observations, they proposed that the fast release of hydrophobic molecules from micelle core into lipid membrane was bridged/facilitated by the high-density poly(ethylene glycol) (PEG) of the micelle’s shell.

Sung’s group developed pH-responsive, Dox-loaded NPs made of *N*-palmitoyl chitosan bearing a Cy5 moiety (Cy5-NPCS) [131]. Caveolae-mediated endocytosis provided the means by which these NPCS NPs were taken up by cells with drug release induced via chitosan breakdown in the acidic endosome/lysosome. By monitoring changes in the FRET signal, they observed that the Dox fluorescence first appeared in the cytosol of cells, while the NPs remained in (slightly acidic) early endosomes. As time passed, and more Dox was released, they observed NPs in late endosomes/lysosomes. Subsequent FRET measurements demonstrated Dox accumulation in the nuclei, with significant cytotoxicity. Such real-time mapping of NP fate with respect to their intracellular localization and drug release is crucial for the rational design of drug carriers.

Chen’s group fabricated multifunctional magnetic nanocarriers for synchronous cancer therapy and sensing [132]. These nanocarriers were synthesized by coupling Dox to adipic, dihydrazide-grafted gum arabic-modified, magnetic nanoparticles (ADH-GAMNP) via pH-sensitive hydrazone bonds. Their ADH-GAMNP-Dox exhibited pH-triggered release of Dox in acidic environments of pH 5.0. The fluorescence of Dox was initially self-quenched via intimate conjugation to GAMNP. Monitoring changes in the fluorescence intensity of Dox-ADH-GAMNP revealed a time and pH-dependent release of Dox.

### 4.3. Mesoporous Silica Nanoparticle-Based Drug Delivery System

Our group has recently developed and evaluated pH-sensitive mesoporous silica nanoparticles (MSNs) for the controlled release of anticancer chemotherapeutics (Figure 5D) [133]. MSNs were synthesized with large pore diameters (~5–7 nm), incorporated the fluorescence dye Atto-647 within their frameworks and had pH-sensitive linkers conjugated onto their nanochannel surfaces via hydrazone bonds. The pH-sensitive linkers for the attachment of Dox possessed hydrazone bonds that were cleavable at endosomal pH. In this configuration, Dox served as the FRET donor and Atto-647, within the MSN’s silica framework, served as the FRET acceptor. To verify the pH-sensitive release of Dox from Atto-647-MSN-hydrazone-Dox, we employed fluorescence spectroscopy and imaging. When Dox remained encapsulated within Atto-647-MSN-hydrazone-Dox, two emission maximal peaks were observed upon excitation of Dox fluorescence at 440 nm: one at 580 nm from Dox alone and the other at 670 nm from Atto-647 that arose from FRET from proximal Dox. Following release of Dox from nanoparticles at pH 4.5, the emission spectra of specimens excited at 440 nm indicated the decrease of fluorescence intensity of both Dox and Atto-647. It was due to the decrease of Dox concentration serving as FRET donor in pH 4.5 acidic buffer. We thereby successfully observed the time-course of pH-responsive release of Dox form our MSNs via measuring changes in FRET.

## 5. Conclusion

In the past decade, remarkable progress has been made in developing nanoparticle-based FRET systems for biosensing, molecular imaging and drug release profiling applications. In this review, we have largely focused upon the utilization of inorganic nanomaterials in the FRET system, such as quantum dots, upconversion nanoparticles, gold nanoparticles and mesoporous silica nanoparticles. But, we have also discussed a few of the more successful polymer-based organic materials for completeness, as well. For their successful application to biological systems, both *in vitro* and *in vivo*, nanoparticle-based FRET probes generally need: (1) strong fluorescence intensity donors, whose emission spectra have significant overlap with the absorption bands of the FRET-pair acceptors’; (2) high stability in the environments in which they operate; (3) intrinsic biocompatibility and/or excretability to minimize adverse local/systemic reactions; and (4) very high targeting specificity, to enhance contrast. If their recent history is any indication of their near future, we can expect to see nanoparticle-based FRET probe development and application accelerating, with their becoming a fundamental tool in biomarker design and drug discovery.
